# Calcium Intake and Associated Factors in a General Japanese Population: Baseline Data of NIPPON DATA80/90 and the National Nutrition Survey

**DOI:** 10.2188/jea.JE20090224

**Published:** 2010-05-05

**Authors:** Mizuka Higashiguchi, Toshiyuki Onoda, Tanvir Chowdhury Turin, Kiyomi Sakata

**Affiliations:** 1 Department of Hygiene and Preventive Medicine, Iwate Medical University, Morioka, Japan; 2 Department of Health Science, Shiga University of Medical Science, Otsu, Japan

**Keywords:** calcium intake, nutrient and food intakes, age group, NIPPON DATA, the National Nutrition Survey

## Abstract

**Objective:**

The purpose of this study was to investigate the dietary calcium intake and associated other dietary factors among representative sample of Japanese general men and women.

**Methods:**

Data was obtained by linking NIPPON DATA80 and 90 with the corresponding National Nutrition Surveys held in 1980 and 1990. We analyzed data for 10 422 subjects (4585 men and 5837 women) in NIPPON DATA80 and 8342 subjects (3488 men and 4854 women) in NIPPON DATA90. Calcium intake was calculated by age groups. Dietary calcium intake was classified into quintiles and physical, life-style, and dietary parameters were examined across the quintiles.

**Results:**

For both men and women, calcium intake tended to be positively associated with age in NIPPON DATA80 and 90, and there were significant differences in estimated calcium intake between age groups. Calcium intake tended to be positively associated with age, protein, fat, saturated fat, vitamins A and C, sodium, potassium, and iron for men and women. Calcium intake also tended to be positively associated with intake of nuts, potatoes, sugar and sweeteners, soybeans and legumes, fruits, green and yellow vegetables, other vegetables, mushrooms, sea algae, fish and shellfish, eggs, and milk and dairy products for men and women.

**Conclusions:**

The characteristics of calcium intake in Japanese people were able to be clarified by using the baseline data of NIPPON DATA and the National Nutrition Survey.

## INTRODUCTION

Calcium, an essential nutrient for human body, plays a crucial role in the regulation of many metabolic processes, in neuromuscular events and in bone health.^1^^,^^2^ Physiologically, calcium is involved in the maintenance of rhythmic contractions of the heart, in blood coagulation, in nerve conduction, in muscle contraction, in the secretion of hormones, in cellular exocytosis, and in cellular adhesion.^1^ Studies have reported the associations of calcium with osteoporosis,^2^^–^^4^ blood pressure,^5^^,^^6^ obesity,^7^^,^^8^ colon cancer,^9^^,^^10^ and cardiovascular diseases (CVD) and stroke.^11^^–^^14^

Insufficient information is available on the dietary calcium intake pattern and its different correlates among Japanese general population. Some more, the entire dietary components needs to be considered when exploring the relationship of chronic diseases conditions and associated risk factors with the dietary patterns of any nutrient in a population, such as dietary intake of calcium that is included in dairy, fish, meat, etc. Calcium intake in Japan is much less than that in Western countries. According to the National Nutrition Survey and the previous studies, the mean calcium intake in Japan was around 450–600 mg/day.^15^^–^^17^ It is assumed that the leading cause of low calcium intake is low milk and dairy products intake. However, detail of the factor that influences the dietary calcium intake is not clear. Accumulation of reliable data to clarify these problems from a general Japanese population is needed to establish strategies for health promotion and prevention of lifestyle-related diseases that take into account the differences between dietary habits in Japan and Western countries.

The purpose of the present study is to obtain necessary fundamental data in relation between the calcium intake and other dietary nutrient and food group among representative sample of Japanese population using data from NIPPON DATA80 and 90 (National Integrated Project for Prospective Observation of Non-communicable Disease And its Trends in the Aged), and the corresponding National Nutrition Survey.

## POPULATIONS AND METHODS

### Nutrition survey

In the National Nutrition Survey before 1995, the dietary intake survey was conducted by a three-day weighed dietary record, and nutrient and food intakes of individuals were calculated by dividing the amount of food intake in a household by the number of household members. Since 1995, nutrient and food intakes of individuals have been calculated by a proportional division method in which the amount of food intake is proportionally divided by the consumption rate of each household member. In this study, we used nutrient and food intakes of individuals in the National Nutrition Survey of 1980 and that of 1990, which were adjusted for average intakes by sex and age groups calculated for the National Nutrition Survey in 1995.^18^^,^^19^ The mean nutrient intake was calculated on the basis of the third revised edition of the Japanese Standard Food Composition Table (which was applied to the National Nutrition Survey in 1980),^20^ and the fourth revised edition (which was applied to the National Nutrition Survey in 1990 and that in 1995).^21^

### Study population

The study sample in this study were participants in two surveys called NIPPON DATA80 (data from the Third National Survey on Circulatory Disorders, Japan in 1980) and NIPPON DATA90 (data from the Fourth National Survey on Circulatory Disorders, Japan in 1990), which were conducted with the National Nutrition Surveys in 1980 and in 1990. NIPPON DATA80 and 90 were performed for all household members aged 30 years or older in 300 census tracts, which were randomly selected throughout Japan. Such data are considered to be representative of the entire population. These surveys have been described in detail previously.^22^^–^^25^ The baseline survey was carried out at local public health centers, and all participants had to be capable of reaching the examination center without assistance. Public health nurses obtained demographic and clinical data from the study participants. Data linkage was performed between corresponding National Nutrition Survey and NIPPON DATA with the objective to investigate fundamental data in relation between the dietary nutritional intake and the health.

### Statistical analysis

After excluding the participants who had missing data or total energy intake of less than 500 kcal or more than 5000 kcal, we analyzed data for the remaining 10 422 subjects (4585 men and 5837 women) in NIPPON DATA80 and 8342 subjects (3488 men and 4854 women) in NIPPON DATA90. All analyses were performed separately for men and women. Calcium intake was calculated as density intake (mg per 1000 kcal). First, average calcium intake was calculated by age groups (aged 30–39, 40–49, 50–59, 60–69 and ≧70 years old). Dietary calcium intake was classified into quintiles and physical, life-style, and dietary parameters were examined across the quintiles. Data are presented as means and standard deviations. Chi-squared tests were used for the categorical variables. To detect differences in continuous variables in groups, analysis of variance (ANOVA) was used. We used SAS Version9.1 (SAS Inc., Cary, NC, USA) for all analyses. Probability values <0.05 were regarded as statistically significant.

## RESULTS

Means of calcium intake by age group are shown in Table 1. From the data obtained from NIPPON DATA80 and 90, the estimated mean values of calcium intake (SD) were 229.3 (60.5) mg/1000 kcal and 240.8 (73.4) mg/1000 kcal, respectively, for men and 275.9 (70.4) mg/1000 kcal and 285.4 (84.9) mg/1000 kcal, respectively, for women. For both men and women, calcium intake tended to be positively associated with age in NIPPON DATA80 and 90, and there were significant differences in estimated calcium intake between age groups.

**Table 1.** Calcium intake by age group in NIPPON DATA80 and 90^a^

	Total	Age group (years)					*P*-value^b^

		30–39	40–49	50–59	60–69	≧70	
NIPPON DATA80							
Men							
*n*	4585	1220	1196	1019	679	471	
Calcium intake (mg/1000 kcal)	229.3 (60.5)	208.7 (51.9)	218.1 (50.9)	236.0 (60.4)	252.6 (65.3)	263.7 (67.3)	<0.001
Women							
*n*	5837	1583	1469	1319	900	566	
Calcium intake (mg/1000 kcal)	275.9 (70.4)	252.8 (56.5)	264.5 (64.1)	293.2 (76.7)	297.7 (74.2)	295.0 (73.5)	<0.001
NIPPON DATA90							
Men							
*n*	3488	660	836	793	708	491	
Calcium intake (mg/1000 kcal)	240.8 (73.4)	210.9 (51.3)	219.8 (58.6)	245.1 (75.2)	258.0 (77.6)	284.7 (82.5)	<0.001
Women							
*n*	4854	1031	1171	1035	915	702	
Calcium intake (mg/1000 kcal)	285.4 (84.9)	252.3 (61.8)	268.4 (73.3)	298.0 (86.9)	314.0 (93.4)	306.3 (94.8)	<0.001

^a^Mean (SD).

^b^Significance of difference was determined by ANOVA.

Tables 2 and 3 show the characteristics of the participants according to quintiles of calcium intake in NIPPON DATA80 and 90, respectively. In both NIPPON DATA80 and 90, calcium intake tended to be positively associated with age, protein, fat, saturated fat, vitamins A and C, sodium, potassium, and iron and tended to be inversely associated with current smoking and drinking habits, total energy, and carbohydrate for men. There were no significant differences in BMI between the five groups of dietary calcium intake for men. On the other hand, for women, calcium intake tended to be positively associated with age, dietary protein, fat, saturated fat, vitamins A and C, sodium, potassium, and iron and tended to be inversely associated with current smoking and drinking habits, total energy, and dietary carbohydrate intake. No significant difference in BMI was observed between the five groups of calcium intake.

**Table 2.** Characteristics of participants according to quintiles of calcium intake in NIPPON DATA80^a^

	Quintiles of calcium intake					*P*-value^b^
	
	Q1	Q2	Q3	Q4	Q5	
Men						
Calcium intake (mg/1000 kcal)	63.7–179.3	179.4–208.5	208.6–237.4	237.5–273.5	273.6–637.2	
*n*	916	917	919	916	917	
Age (years)	45.1 (11.9)	46.8 (12.0)	49.3 (13.0)	51.7 (13.1)	56.8 (13.7)	<0.001
Body mass index (kg/m^2^)	22.5 (2.8)	22.6 (2.8)	22.5 (2.9)	22.6 (2.9)	22.4 (2.9)	0.42
Total cholesterol (mg/dL)	185.9 (32.7)	187.4 (33.0)	186.4 (32.3)	184.1 (31.9)	187.9 (34.1)	0.12
Systolic blood pressure (mm Hg)	135.8 (20.5)	136.4 (21.1)	137.8 (20.7)	139.5 (21.1)	142.4 (21.3)	<0.001
Diastolic blood pressure (mm Hg)	82.9 (12.6)	83.0 (12.3)	83.8 (12.3)	84.1 (12.3)	83.8 (12.4)	0.15
Current smoker (%)	69.4	65.4	62.8	59.8	58.3	<0.001
Current drinker (%)	77.4	78.1	74.2	73.2	68.9	<0.001
Total energy (kcal)	2461.1 (528.4)	2444.4 (485.3)	2409.3 (481.1)	2415.6 (472.0)	2275.9 (521.3)	<0.001
Carbohydrate (%kcal)	60.6 (6.4)	60.1 (6.1)	59.9 (6.2)	59.3 (6.3)	58.8 (7.1)	<0.001
Protein (%kcal)	13.8 (1.6)	14.5 (1.7)	15.1 (1.8)	15.5 (1.9)	16.5 (2.3)	<0.001
Fat (%kcal)	19.5 (5.6)	19.9 (5.1)	19.8 (4.9)	20.2 (5.2)	20.5 (5.2)	<0.001
Saturated fat (%kcal)	5.3 (1.3)	5.6 (1.4)	5.6 (1.3)	5.8 (1.5)	6.0 (1.7)	0.001
Vitamin A (mg/1000 kcal)	519.7 (198.4)	648.0 (221.5)	721.6 (256.2)	800.5 (301.0)	942.7 (392.9)	<0.001
Vitamin C (mg/1000 kcal)	33.9 (11.6)	41.1 (13.2)	46.0 (14.6)	50.7 (16.2)	59.8 (22.4)	<0.001
Sodium (mg/1000 kcal)	2075.3 (630.0)	2346.9 (653.3)	2478.3 (723.6)	2662.0 (834.3)	2889.0 (1000.0)	<0.001
Potassium (mg/1000 kcal)	1042.7 (147.1)	1170.8 (144.0)	1262.1 (143.2)	1363.7 (171.0)	1532.8 (237.0)	<0.001
Iron (mg/1000 kcal)	5.3 (0.8)	5.9 (0.7)	6.3 (0.8)	6.7 (0.9)	7.4 (1.3)	<0.001
Women						
Calcium intake (mg/1000 kcal)	86.3–217.3	217.4–250.8	250.9–284.9	285.0–328.8	328.9–731.2	
*n*	1166	1169	1167	1167	1168	
Age (years)	46.1 (12.9)	47.2 (13.1)	49.7 (13.4)	51.9 (13.2)	55.4 (12.8)	<0.001
Body mass index (kg/m^2^)	22.8 (3.5)	22.6 (3.3)	22.8 (3.4)	22.9 (3.2)	23.0 (3.3)	0.10
Total cholesterol (mg/dL)	186.0 (32.9)	188.4 (33.9)	190.3 (34.4)	193.1 (33.4)	198.2 (34.1)	<0.001
Systolic blood pressure (mm Hg)	131.3 (21.4)	131.8 (21.1)	133.3 (21.8)	135.4 (21.5)	137.4 (22.3)	<0.001
Diastolic blood pressure (mm Hg)	78.6 (12.2)	78.7 (11.8)	79.6 (12.7)	80.1 (11.2)	80.8 (11.9)	<0.001
Current smoker (%)	12.2	10.9	9.3	8.7	8.1	0.041
Current drinker (%)	21.8	23.5	20.5	18.7	17.3	0.002
Total energy (kcal)	1982.3 (428.7)	1946.9 (377.5)	1935.1 (931.4)	1927.8 (368.5)	1854.9 (431.7)	<0.001
Carbohydrate (%kcal)	63.3 (6.8)	62.3 (6.8)	62.1 (6.7)	61.8 (6.8)	60.9 (6.8)	<0.001
Protein (%kcal)	14.1 (1.6)	14.9 (1.8)	15.4 (1.8)	15.9 (1.9)	17.0 (2.2)	<0.001
Fat (%kcal)	20.9 (6.0)	21.7 (5.8)	21.6 (5.6)	21.9 (5.6)	22.4 (5.7)	<0.001
Saturated fat (%kcal)	5.7 (1.5)	6.1 (1.6)	6.2 (1.6)	6.3 (1.7)	6.6 (1.8)	<0.001
Vitamin A (mg/1000 kcal)	610.0 (242.4)	753.5 (257.8)	843.1 (306.6)	942.7 (352.7)	1134.4 (473.8)	<0.001
Vitamin C (mg/1000 kcal)	45.2 (15.7)	54.3 (17.6)	60.2 (18.8)	67.4 (22.4)	79.3 (28.8)	<0.001
Sodium (mg/1000 kcal)	2246.8 (662.3)	2520.1 (727.6)	2632.2 (723.9)	2810.9 (796.3)	3111.6 (1082.7)	<0.001
Potassium (mg/1000 kcal)	1178.7 (170.7)	1328.8 (170.9)	1423.9 (178.0)	1537.0 (198.1)	1745.2 (273.3)	<0.001
Iron (mg/1000 kcal)	5.9 (0.8)	6.5 (0.9)	6.9 (1.0)	7.5 (1.0)	8.3 (1.4)	<0.001

^a^Mean (SD).

^b^Significance of difference was determined by ANOVA or chi-square test.

**Table 3.** Characteristics of participants according to quintiles of calcium intake in NIPPON DATA90^a^

	Quintiles of calcium intake					*P*-value^b^
	
	Q1	Q2	Q3	Q4	Q5	
Men						
Calcium intake (mg/1000 kcal)	76.3–181.6	181.7–214.0	214.1–245.0	245.1–292.2	292.3–728.8	
*n*	697	698	698	698	697	
Age (years)	48.4 (12.4)	49.4 (12.6)	51.8 (13.3)	56.0 (14.0)	60.8 (12.5)	<0.001
Body mass index (kg/m^2^)	22.9 (3.0)	23.1 (3.1)	22.9 (2.8)	22.9 (3.0)	22.8 (3.1)	0.53
Total cholesterol (mg/dL)	196.2 (36.5)	199.0 (35.9)	197.8 (36.6)	199.4 (36.8)	200.7 (38.3)	0.24
Systolic blood pressure (mm Hg)	135.6 (20.3)	135.6 (19.1)	136.6 (20.0)	138.6 (19.5)	142.0 (20.4)	<0.001
Diastolic blood pressure (mm Hg)	83.1 (11.7)	83.2 (11.3)	83.2 (11.9)	84.0 (11.2)	84.2 (12.1)	0.23
Current smoker (%)	64.0	62.7	54.8	49.7	43.8	<0.001
Current drinker (%)	61.4	61.7	58.4	53.8	53.3	<0.001
Total energy (kcal)	2362.4 (493.7)	2355.5 (451.6)	2315.5 (430.7)	2289.6 (456.3)	2256.5 (465.1)	<0.001
Carbohydrate (%kcal)	57.6 (6.2)	56.8 (5.4)	56.8 (5.6)	56.4 (6.1)	56.0 (5.5)	<0.001
Protein (%kcal)	14.6 (1.9)	15.0 (1.6)	15.5 (1.7)	16.1 (1.9)	16.7 (1.9)	<0.001
Fat (%kcal)	21.5 (4.8)	22.3 (4.3)	22.3 (4.4)	22.7 (4.4)	22.9 (4.4)	<0.001
Saturated fat (%kcal)	5.4 (1.3)	5.7 (1.2)	5.9 (1.3)	6.1 (1.4)	6.4 (1.4)	<0.001
Vitamin A (mg/1000 kcal)	981.0 (1029.8)	1119.1 (1041.0)	1236.4 (1116.0)	1393.3 (1273.2)	1543.0 (1465.3)	<0.001
Vitamin C (mg/1000 kcal)	41.0 (19.8)	51.0 (30.5)	54.7 (23.9)	60.1 (21.6)	69.4 (25.4)	<0.001
Sodium (mg/1000 kcal)	2292.7 (601.2)	2463.1 (641.7)	2582.3 (758.3)	2551.8 (693.2)	2641.7 (718.4)	<0.001
Potassium (mg/1000 kcal)	1092.1 (171.0)	1214.5 (179.0)	1304.8 (186.1)	1402.1 (222.8)	1568.8 (264.4)	<0.001
Iron (mg/1000 kcal)	4.7 (0.7)	5.2 (0.8)	5.5 (0.8)	5.8 (0.9)	6.5 (1.4)	<0.001
Women						
Calcium intake (mg/1000 kcal)	90.9–217.6	217.7–254.3	254.4–291.1	291.2–345.9	346.0–888.4	
*n*	970	970	971	971	971	
Age (years)	48.8 (13.7)	50.1 (13.9)	51.6 (14.3)	54.9 (13.8)	58.6 (12.6)	<0.001
Body mass index (kg/m^2^)	22.8 (3.4)	22.9 (3.4)	22.9 (3.3)	22.7 (3.2)	22.8 (3.3)	0.85
Total cholesterol (mg/dL)	201.6 (37.2)	201.2 (38.8)	204.2 (37.1)	211.5 (38.4)	216.4 (40.5)	<0.001
Systolic blood pressure (mm Hg)	132.2 (21.6)	131.4 (20.7)	132.0 (19.9)	135.3 (21.2)	137.5 (20.0)	<0.001
Diastolic blood pressure (mm Hg)	79.2 (12.1)	78.7 (11.9)	78.7 (11.6)	80.0 (11.4)	81.1 (11.6)	<0.001
Current smoker (%)	14.8	9.9	9.4	7.2	6.3	<0.001
Current drinker (%)	7.4	8.3	6.6	5.9	4.4	0.016
Total energy (kcal)	1877.8 (382.9)	1871.9 (367.6)	1853.2 (338.6)	1866.0 (358.9)	1824.9 (382.0)	0.013
Carbohydrate (%kcal)	59.7 (6.6)	59.3 (6.2)	58.8 (6.1)	58.4 (5.8)	58.3 (5.8)	<0.001
Protein (%kcal)	14.9 (1.9)	15.5 (1.7)	15.8 (1.7)	16.4 (1.9)	17.2 (2.0)	<0.001
Fat (%kcal)	23.7 (5.4)	24.1 (5.1)	24.6 (5.0)	24.7 (4.7)	24.9 (4.8)	<0.001
Saturated fat (%kcal)	5.9 (1.4)	6.2 (1.4)	6.5 (1.5)	6.7 (1.5)	7.0 (1.6)	<0.001
Vitamin A (mg/1000 kcal)	1123.5 (1068.8)	1288.6 (1167.8)	1424.5 (1700.6)	1599.7 (1471.7)	1737.3 (1265.6)	<0.001
Vitamin C (mg/1000 kcal)	54.3 (23.4)	65.0 (26.1)	71.4 (38.4)	77.2 (29.1)	88.4 (32.2)	<0.001
Sodium (mg/1000 kcal)	2530.8 (682.5)	2652.4 (668.4)	2746.7 (778.6)	2763.4 (787.0)	2925.0 (884.6)	<0.001
Potassium (mg/1000 kcal)	1233.7 (198.2)	1376.1 (208.5)	1464.2 (220.1)	1571.2 (242.4)	1784.6 (310.0)	<0.001
Iron (mg/1000 kcal)	5.2 (0.8)	5.7 (0.8)	5.9 (0.9)	6.3 (1.0)	7.1 (1.6)	<0.001

^a^Mean (SD).

^b^Significance of difference was determined by ANOVA or chi-square test.

Tables 4 and 5 show the food group intakes according to quintiles of calcium intake in NIPPON DATA80 and 90, respectively. In both NIPPON DATA80 and 90, calcium intake tended to be positively associated with intake of nuts, potatoes, sugar and sweeteners, sweets and snacks, soybeans and legumes, fruits, green and yellow vegetables, other vegetables, mushrooms, sea algae, fish and shellfish, eggs, and milk and dairy products and tended to be inversely associated with intake of cereals, rice, fats and oils, and meats for men. There were no significant differences in condiments and beverages in NIPPON DATA80 and flour products in NIPPON DATA90 between the five groups of calcium intake. On the other hand, calcium intake tended to be positively associated with intake of nuts, potatoes, sugar and sweeteners, soybeans and legumes, fruits, green and yellow vegetables, other vegetables, mushrooms, sea algae, fish and shellfish, eggs, and milk and dairy products and tended to be inversely associated with intake of cereals, rice, flour products, fats and oils, and meats for women. There were no significant differences in condiments and beverages in NIPPON DATA80 and sweets and snacks in NIPPON DATA90 between the five groups of calcium intake.

**Table 4.** Food intakes according to quintiles of calcium intake in NIPPON DATA80^a^

	Quintiles of calcium intake					*P*-value^b^
	
	Q1	Q2	Q3	Q4	Q5	
Men						
Calcium intake (mg/1000 kcal)	63.7–179.3	179.4–208.5	208.6–237.4	237.5–273.5	273.6–637.2	
*n*	916	917	919	916	917	
Cereals (g/1000 kcal)	177.1 (29.8)	166.9 (27.4)	160.5 (27.1)	152.0 (26.8)	144.5 (30.9)	<0.001
Rice (g/1000 kcal)	142.0 (34.8)	131.3 (32.4)	125.5 (32.0)	120.7 (32.2)	122.4 (34.5)	<0.001
Flour product (g/1000 kcal)	38.8 (27.6)	38.8 (24.5)	37.4 (24.1)	33.7 (23.7)	33.5 (25.2)	<0.001
Nuts (g/1000 kcal)	0.3 (1.2)	0.5 (1.6)	0.5 (1.6)	0.6 (2.1)	0.8 (2.0)	<0.001
Potatoes (g/1000 kcal)	22.2 (16.8)	25.5 (17.3)	27.8 (18.2)	30.5 (20.3)	32.4 (21.7)	<0.001
Sugar and sweeteners (g/1000 kcal)	5.2 (4.0)	5.6 (4.0)	5.8 (4.2)	5.9 (4.1)	6.0 (4.1)	<0.001
Sweets and snacks (g/1000 kcal)	5.1 (6.0)	5.9 (5.9)	6.7 (7.3)	6.5 (7.1)	7.5 (9.0)	<0.001
Fats and oils (g/1000 kcal)	7.8 (4.8)	7.6 (4.4)	6.9 (4.1)	6.9 (4.3)	6.3 (4.0)	<0.001
Soybeans and legumes (g/1000 kcal)	21.1 (13.3)	29.1 (14.6)	34.0 (16.7)	39.8 (19.4)	49.4 (27.0)	<0.001
Fruits (g/1000 kcal)	39.9 (30.1)	52.9 (34.1)	58.6 (35.6)	65.7 (38.3)	78.9 (47.6)	<0.001
Green and yellow vegetables (g/1000 kcal)	15.8 (10.4)	20.5 (12.7)	23.9 (13.9)	26.4 (17.0)	32.4 (21.5)	<0.001
Other vegetables (g/1000 kcal)	78.0 (28.6)	87.9 (29.8)	95.2 (34.1)	104.4 (38.5)	117.8 (49.4)	<0.001
Mushrooms (g/1000 kcal)	3.7 (5.2)	3.7 (5.0)	4.1 (5.3)	4.2 (5.2)	5.0 (6.3)	<0.001
Sea algae (g/1000 kcal)	1.3 (1.3)	1.8 (1.9)	2.3 (2.3)	3.0 (2.8)	5.1 (5.7)	<0.001
Condiments and beverages (g/1000 kcal)	81.8 (86.6)	82.3 (72.1)	82.3 (71.5)	86.9 (74.2)	78.1 (71.0)	0.17
Fish and shellfish (g/1000 kcal)	46.4 (22.5)	48.5 (22.9)	52.4 (23.8)	54.4 (25.9)	59.8 (29.7)	<0.001
Meats (g/1000 kcal)	32.9 (17.0)	31.7 (17.2)	29.3 (15.3)	28.1 (15.6)	25.5 (16.3)	<0.001
Eggs (g/1000 kcal)	15.3 (9.3)	17.1 (8.5)	17.1 (9.2)	17.9 (9.2)	18.6 (11.3)	<0.001
Milk and dairy products (g/1000 kcal)	14.9 (11.4)	22.0 (14.2)	28.4 (17.0)	35.3 (21.9)	51.1 (35.6)	<0.001
Women						
Calcium intake (mg/1000 kcal)	86.3–217.3	217.4–250.8	250.9–284.9	285.0–328.8	328.9–731.2	
*n*	1166	1169	1167	1167	1168	
Cereals (g/1000 kcal)	170.7 (27.9)	160.0 (28.2)	155.1 (26.2)	148.0 (26.7)	138.6 (29.5)	<0.001
Rice (g/1000 kcal)	123.2 (32.1)	111.2 (32.2)	108.0 (31.2)	105.6 (31.7)	97.8 (31.8)	<0.001
Flour product (g/1000 kcal)	45.0 (28.5)	47.6 (30.1)	45.6 (29.2)	41.2 (27.4)	39.7 (28.9)	<0.001
Nuts (g/1000 kcal)	0.5 (2.0)	0.8 (2.6)	0.9 (2.8)	0.9 (2.3)	1.2 (2.8)	<0.001
Potatoes (g/1000 kcal)	27.6 (20.7)	31.6 (21.0)	33.3 (21.6)	35.7 (24.1)	39.4 (27.7)	<0.001
Sugar and sweeteners (g/1000 kcal)	6.5 (5.0)	7.2 (5.1)	6.8 (4.7)	6.7 (4.6)	6.7 (4.5)	<0.001
Sweets and snacks (g/1000 kcal)	11.7 (13.1)	13.5 (13.6)	14.2 (13.6)	13.9 (13.9)	13.4 (14.1)	<0.001
Fats and oils (g/1000 kcal)	8.3 (4.9)	8.6 (4.9)	8.0 (4.6)	7.7 (4.6)	7.3 (4.7)	<0.001
Soybeans and legumes (g/1000 kcal)	24.1 (14.3)	29.8 (15.4)	36.4 (17.9)	43.8 (21.8)	52.6 (28.5)	<0.001
Fruits (g/1000 kcal)	68.6 (48.7)	86.3 (51.3)	95.3 (51.8)	105.9 (59.7)	124.2 (72.3)	<0.001
Green and yellow vegetables (g/1000 kcal)	20.2 (13.8)	25.8 (15.5)	30.1 (18.0)	34.3 (20.6)	43.2 (28.5)	<0.001
Other vegetables (g/1000 kcal)	88.3 (33.2)	100.7 (35.9)	107.4 (39.0)	118.3 (43.0)	132.9 (54.2)	<0.001
Mushrooms (g/1000 kcal)	4.0 (5.6)	4.4 (5.8)	4.4 (5.5)	4.9 (6.1)	5.7 (7.2)	<0.001
Sea algae (g/1000 kcal)	1.5 (1.6)	2.1 (2.1)	2.6 (2.8)	3.6 (3.5)	6.2 (6.9)	<0.001
Condiments and beverages (g/1000 kcal)	34.7 (47.5)	35.6 (35.9)	34.7 (31.7)	36.0 (32.3)	34.8 (50.8)	0.91
Fish and shellfish (g/1000 kcal)	44.1 (21.9)	47.3 (23.1)	50.3 (23.7)	51.8 (23.7)	58.2 (27.8)	<0.001
Meats (g/1000 kcal)	30.1 (16.6)	30.0 (16.7)	27.8 (15.3)	27.0 (14.9)	24.8 (15.4)	<0.001
Eggs (g/1000 kcal)	16.6 (9.7)	18.2 (10.3)	18.5 (9.6)	18.7 (9.8)	19.8 (11.7)	<0.001
Milk and dairy products (g/1000 kcal)	24.0 (18.1)	37.7 (23.4)	45.7 (28.6)	53.8 (32.7)	75.9 (50.2)	<0.001

^a^Mean (SD).

^b^Significance of difference was determined by ANOVA.

**Table 5.** Food intakes according to quintiles of calcium intake in NIPPON DATA90^a^

	Quintiles of calcium intake					*P*-value^b^
	
	Q1	Q2	Q3	Q4	Q5	
Men						
Calcium intake (mg/1000 kcal)	76.3–181.6	181.7–214.0	214.1–245.0	245.1–292.2	292.3–728.8	
*n*	697	698	698	698	697	
Cereals (g/1000 kcal)	161.2 (28.2)	151.3 (26.8)	146.6 (24.5)	141.9 (26.9)	132.9 (27.2)	<0.001
Rice (g/1000 kcal)	127.5 (31.6)	116.5 (29.5)	113.6 (29.1)	106.1 (27.6)	96.6 (27.8)	<0.001
Flour product (g/1000 kcal)	36.0 (26.7)	36.6 (23.9)	34.7 (23.7)	36.3 (23.5)	35.9 (26.2)	0.67
Nuts (g/1000 kcal)	0.4 (1.3)	0.5 (1.4)	0.6 (1.9)	0.8 (1.8)	1.1 (2.2)	<0.001
Potatoes (g/1000 kcal)	25.6 (16.2)	27.8 (17.3)	29.4 (17.6)	31.2 (19.9)	34.0 (20.5)	<0.001
Sugar and sweeteners (g/1000 kcal)	4.8 (3.7)	5.2 (3.6)	5.4 (3.4)	5.3 (3.9)	5.6 (4.2)	0.001
Sweets and snacks (g/1000 kcal)	4.8 (6.6)	5.7 (6.7)	5.6 (7.2)	6.4 (8.4)	6.3 (8.4)	<0.001
Fats and oils (g/1000 kcal)	7.9 (4.4)	8.1 (4.0)	7.6 (3.8)	7.2 (3.7)	6.6 (3.7)	<0.001
Soybeans and legumes (g/1000 kcal)	26.8 (15.6)	32.9 (17.7)	38.1 (19.2)	41.6 (21.4)	48.6 (27.0)	<0.001
Fruits (g/1000 kcal)	35.0 (33.1)	44.7 (33.6)	51.8 (37.4)	60.8 (40.0)	72.6 (47.1)	<0.001
Green and yellow vegetables (g/1000 kcal)	26.5 (16.4)	31.8 (17.1)	36.5 (18.2)	41.7 (21.6)	52.8 (31.3)	<0.001
Other vegetables (g/1000 kcal)	70.0 (28.0)	78.4 (31.1)	84.5 (32.7)	90.6 (36.0)	91.9 (38.1)	<0.001
Mushrooms (g/1000 kcal)	4.3 (5.5)	4.9 (5.5)	5.1 (5.8)	6.0 (7.0)	6.7 (7.6)	<0.001
Sea algae (g/1000 kcal)	2.2 (2.8)	2.7 (2.8)	3.2 (3.5)	3.8 (5.1)	4.8 (5.6)	<0.001
Condiments and beverages (g/1000 kcal)	108.4 (97.1)	123.9 (111.8)	107.6 (86.9)	93.6 (78.5)	89.9 (80.5)	<0.001
Fish and shellfish (g/1000 kcal)	49.9 (24.9)	52.1 (22.1)	55.8 (24.7)	57.5 (26.5)	59.6 (23.9)	<0.001
Meats (g/1000 kcal)	34.1 (17.5)	32.4 (15.5)	30.9 (15.7)	29.4 (16.1)	25.3 (14.3)	<0.001
Eggs (g/1000 kcal)	18.3 (9.8)	19.5 (9.5)	19.4 (9.6)	20.6 (9.7)	20.8 (9.8)	<0.001
Milk and dairy products (g/1000 kcal)	14.9 (12.9)	25.3 (16.4)	36.1 (20.0)	49.4 (25.7)	73.8 (47.9)	<0.001
Women						
Calcium intake (mg/1000 kcal)	90.9–217.6	217.7–254.3	254.4–291.1	291.2–345.9	346.0–888.4	
*n*	970	970	971	971	971	
Cereals (g/1000 kcal)	154.4 (26.9)	147.4 (26.2)	141.3 (24.9)	136.2 (24.3)	128.4 (32.4)	<0.001
Rice (g/1000 kcal)	110.3 (30.3)	101.9 (28.1)	97.0 (28.3)	93.3 (26.1)	87.4 (30.7)	<0.001
Flour product (g/1000 kcal)	41.5 (27.8)	43.7 (26.8)	43.0 (26.7)	41.6 (26.4)	39.4 (27.5)	0.006
Nuts (g/1000 kcal)	0.6 (2.0)	0.6 (1.6)	0.8 (2.1)	1.0 (2.5)	1.3 (2.6)	<0.001
Potatoes (g/1000 kcal)	30.9 (20.7)	33.9 (21.6)	35.4 (21.5)	37.3 (22.6)	40.8 (24.9)	<0.001
Sugar and sweeteners (g/1000 kcal)	5.9 (4.3)	6.3 (4.6)	6.5 (4.3)	6.2 (4.6)	6.5 (4.7)	0.011
Sweets and snacks (g/1000 kcal)	11.0 (13.7)	12.0 (13.3)	11.8 (13.5)	12.1 (13.2)	11.5 (13.9)	0.39
Fats and oils (g/1000 kcal)	9.1 (4.9)	8.9 (4.6)	8.8 (4.6)	8.1 (4.0)	7.5 (4.2)	<0.001
Soybeans and legumes (g/1000 kcal)	28.9 (16.9)	35.4 (19.6)	39.0 (20.2)	43.4 (22.8)	51.4 (27.8)	<0.001
Fruits (g/1000 kcal)	57.5 (46.4)	74.5 (53.6)	83.0 (55.8)	91.1 (57.2)	106.2 (60.9)	<0.001
Green and yellow vegetables (g/1000 kcal)	33.7 (21.2)	40.1 (21.8)	44.1 (22.9)	52.5 (27.0)	66.0 (36.1)	<0.001
Other vegetables (g/1000 kcal)	80.5 (33.3)	90.7 (35.8)	95.7 (37.1)	99.7 (39.5)	104.2 (43.6)	<0.001
Mushrooms (g/1000 kcal)	5.1 (7.4)	5.5 (6.1)	5.9 (6.2)	6.6 (7.2)	7.7 (8.8)	<0.001
Sea algae (g/1000 kcal)	2.6 (3.4)	3.2 (3.2)	3.7 (3.9)	4.4 (5.9)	5.8 (6.8)	<0.001
Condiments and beverages (g/1000 kcal)	46.7 (48.2)	46.4 (40.5)	47.3 (41.7)	43.2 (37.2)	40.1 (32.4)	<0.001
Fish and shellfish (g/1000 kcal)	48.1 (22.7)	51.3 (21.7)	52.5 (22.7)	55.4 (24.8)	57.3 (23.3)	<0.001
Meats (g/1000 kcal)	32.7 (17.3)	30.5 (14.9)	29.8 (15.3)	28.4 (15.6)	25.1 (14.3)	<0.001
Eggs (g/1000 kcal)	19.8 (10.7)	21.0 (10.7)	20.9 (9.9)	21.7 (10.2)	22.0 (10.7)	<0.001
Milk and dairy products (g/1000 kcal)	23.2 (19.5)	41.3 (26.1)	55.7 (32.3)	74.4 (36.7)	102.6 (57.6)	<0.001

^a^Mean (SD).

^b^Significance of difference was determined by ANOVA.

## DISCUSSION

In this cross-sectional study in representative Japanese men and women, we investigated calcium intake and other associated dietary factors. For men and women, higher calcium intake was associated with more advanced age. Calcium intake tended to be inversely associated with intake of meats and it seemed to be related to more advanced age. Nevertheless, calcium intake tended to be positively associated with intake of protein, fat, and saturated fat, and these results seem to be related to higher intake of soybeans and legumes, fish and shellfish, eggs, and milk and dairy products. Calcium intake tended to be positively associated with intake of vitamins A and C, and potassium and iron, and it seemed to be related to higher intake of fruits, green and yellow vegetables, other vegetables, mushrooms, and sea algae. We observed that the participants with higher intake of dietary calcium tended to be from older age categories. These findings are comparable to the National Nutrition Survey in 2003 findings,^15^ which demonstrated that calcium intake of people aged 50 and over was higher than younger people. The National Nutrition Survey in 2003 was based on the individual nutrition intake data. The INTERMAP study that based on 24-hour dietary recall and involved people of 40–59 years of age from four centres in Japan reported lower intake of calcium among younger age group people.^16^ The Japan Collaborative Cohort Study (JACC Study) that was based on food frequency questionnaire and involved people of 40–79 years of age from 45 communities across Japan also reported similar findings.^17^ Direct comparison is not possible because of differences in the method and period of the dietary survey in the present study and the previous studies. Dietary record is considered to be the gold standard to assess nutrition survey because it has high validity and reliability. Even if it is taken into consideration that calcium intake was adjusted for average intakes by sex and age groups calculated for the National Nutrition Survey in 1995, the results of this study that was calculated by three-day weighing record method in the National Nutrition Survey are not likely to compare unfavorably with the findings in the previous studies in validity and reliability.

Calcium intake in Japan is less than that in Western countries. According to the INTERMAP study, levels of calcium intake per day for people of 40–59 years of age were 1013.3 mg for men and 843.1 mg for women in the United Kingdom and 882.2 mg for men and 699.5 mg for women in the United States, whereas the levels were only 605.4 mg for men and 606.9 mg for women in Japan.^16^ Although direct comparison is not possible in the present study and the INTERMAP study, we also found that calcium intake in Japan is lower than that in Western countries. A guideline for dietary intake of calcium was set by the Ministry of Health, Labour and Welfare, the Ministry of Agriculture, Forestry and Fisheries, and the Ministry of Education, Culture, Sports, Science and Technology in 2000.^26^ Milk and dairy products, legumes, green and yellow vegetables intakes are recommended to increase the calcium intake by this guideline. In National Health Promotion in the 21st Century (Health Japan 21) that started in 2000, daily levels of food intake for sufficient calcium supply, such as more than 130 g for milk and dairy products, more than 100 g for legumes and more than 120 g for green and yellow vegetables,^27^ have been set, but current intake levels of 125.1 g, 59.3 g and 94.4 g, respectively,^28^ are below these levels. In the past couple of decades, there has been almost no change in calcium intake, as indicated by data obtained in NIPPON DATA80, NIPPON DATA90 and the National Health and Nutrition Survey in 2005.^28^

Recent studies have shown that calcium intake, especially calcium intake from milk and dairy products, has a protective effect against obesity^7^^,^^8^ and stroke.^11^^–^^14^ Milk and dairy products include many functional components (eg, linoleic acid, sphingolipids, and milk protein),^29^^–^^31^ and availability of calcium for absorption by the intestine is high.^32^ In this study, higher calcium intake was associated with higher intake of milk and dairy products, but intake of milk and dairy products in Japan is far lower than that in Western countries.^33^ We also found that higher calcium intake was associated with higher intake of nuts, soybeans and legumes, green and yellow vegetables, and other vegetables, but these foods contain substances that are likely to inhibit the intestinal absorption of calcium, such as phytates, oxalates, tannin and uronic acids.^34^ Thus, it is necessary not only to investigate total calcium intake but also to estimate the calcium supply rate from each food and to consider the difference in the ratio of intestinal calcium absorption and effects of components other than calcium.

In this study, information on food sources such as regular foods, enriched/fortified foods, and supplements was not available. However, the use of calcium-enriched/fortified foods and supplements is infrequent in Japan. According to the National Health and Nutrition Survey in 2006, 3.7% of Japanese people used calcium-enriched/fortified foods and supplements and the average supply rate of calcium from these foods is only 0.9%,^35^ and it is thought to be lower in 1980 and 1990. Additionally, we were not able to obtain information on pregnancy, lactation and menopause statuses of the female subjects.

In conclusion, dietary calcium intake is likely to be associated with intake of other dietary nutrients and various foods, and it seemed to be characterized by the dietary patterns of a person. To establish strategies for prevention of lifestyle-related diseases such as osteoporosis, colon cancer and CVD, it is necessary to accumulate reliable data from Japanese people because calcium intake is different between Japan and Western countries. We obtained necessary fundamental data to investigate the relation between the calcium intake and the risk factors of these diseases.
